# Metal-Based Regenerative Strategies for Peripheral Nerve Injuries: From Biodegradable Ion Source to Stable Conductive Implants

**DOI:** 10.34133/bmr.0219

**Published:** 2025-07-22

**Authors:** Hyewon Kim, Khandoker Asiqur Rahaman, Jieun Kwon, Seohyeon Cho, Seok Chung, Hyung-Seop Han, Yu-Chan Kim

**Affiliations:** ^1^ Biomaterials Research Center, Biomedical Research Division, Korea Institute of Science and Technology (KIST), Seoul 02792, Republic of Korea.; ^2^ Department of Biomicro System Technology, Korea University, Seoul 02841, Republic of Korea.; ^3^ Department of Materials Science & Engineering, Seoul National University, Seoul 08826, Republic of Korea.; ^4^ Division of Bio-Medical Science & Technology, KIST School, University of Science and Technology (UST), Seoul 02792, Republic of Korea.

## Abstract

Peripheral nerve injury is a common health issue in modern aging societies, with the only treatment available being autograft transplantation. Unfortunately, autograft is often limited due to donor availability and immune rejection. Additionally, the peripheral nervous system has limited regenerative capacity, making the treatment of peripheral nerve injuries challenging. Metal-based regenerative medicine and tissue engineering strategies provide advanced solutions to the problem. Metal-based biomaterials such as conduits, filaments, alloys, hydrogels, and ceramics can deliver biofunctional metal ions and promote axonal growth and functional recovery. In parallel, metal-based electromagnetic stimulation demonstrates potential for nerve regeneration and inflammation regulation. The potential of metal-based biomaterials in promoting peripheral nerve regeneration highlights the need for further research in tissue engineering and regenerative medicine. However, rapid degradation, long-term biocompatibility, and necessary optimization regarding injury types remain to be explored. This review summarizes the reported metal-based biomaterials utilized in peripheral nerve regeneration research. The aim is to showcase advanced technologies available in the field, which may potentially become a viable alternative to autografts, offering transformative applications in the regenerative medical field.

## Introduction

**Fig. 1.**
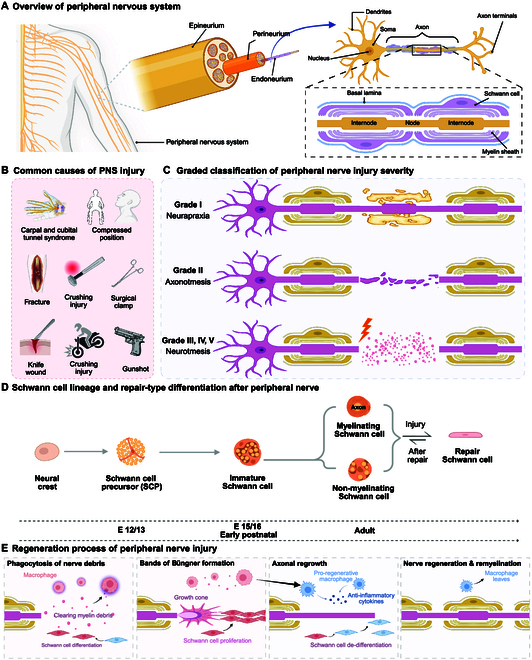
Structural organization, classification, cellular dynamics, and regeneration process of PNI. (A) Schematic representation of the peripheral nervous system anatomy, highlighting the hierarchical organization of connective tissue layers, which are epineurium, perineurium, and endoneurium surrounding individual nerve fascicles. The microarchitecture includes axons ensheathed by Schwann cells and organized into myelinated internodal segments. (B) Common etiologies of PNIs include mechanical compression (e.g., carpal or cubital tunnel syndrome), blunt trauma (e.g., fractures and crush injuries), and penetrating injuries (e.g., surgical clamps, gunshot wounds, and knife lacerations). (C) Graded classification of PNI severity based on Sunderland’s scale, ranging from grade I (neuropraxia: transient conduction block without axonal damage), grade II (axonotmesis: axonal disruption with preserved connective tissue architecture), to grades III to V (neurotmesis: progressive structural damage involving endoneurial, perineurial, and epineurial disruption). (D) Developmental lineage and injury-induced plasticity of Schwann cells. Schwann cells arise from neural crest cells via a precursor stage and differentiate into myelinating or nonmyelinating phenotypes. Upon injury, mature Schwann cells dedifferentiate into a repair phenotype, enabling axonal guidance, debris clearance, and trophic support, followed by redifferentiation during regeneration. (E) Sequential events during peripheral nerve regeneration, including macrophage-mediated debris clearance, Schwann cell reprogramming and proliferation, formation of bands of Büngner, axonal elongation, and eventual remyelination, culminating in functional restoration of nerve integrity.

Peripheral nerve injuries (PNIs) present a significant clinical challenge (Fig. 1A), often leading to incomplete recovery and long-term disability due to the limited regenerative capacity of the nervous system [1]. Recent epidemiological studies indicate that the incidence of PNIs varies globally (Fig. 1B). For instance, a national Swedish survey reported decreased PNI incidence from 18.6 to 12.9 per 100,000 individuals between 2008 and 2022, with higher rates observed in men than in women [2]. In South Korea, the annual incidence of upper extremity PNIs decreased from 10.67 to 7.88 per 100,000 persons between 2008 and 2018 [3]. Despite these trends, PNIs remain prevalent, particularly among working-age males, and are often associated with traumatic events such as motor vehicle accidents and occupational injuries [4].

PNIs are challenging to treat due to the nervous system’s limited regenerative capacity, often resulting in incomplete recovery and long-term disability [5]. This slow and inadequate healing process underscores the need for more advanced and effective therapeutic approaches. Autografts, allografts, and synthetic conduits are traditional treatments that have been researched [6]. However, their ability to fully restore nerve function is limited and not free from complications, requiring the investigation of novel approaches [7]. Metal-based biomaterials have demonstrated potential in the field of regenerative medicine, particularly in the area of peripheral nerve regeneration [8]. These materials can deliver biofunctional ions such as magnesium, calcium, zinc, iron, and lithium [9–13], which are known to support nerve repair through various biological mechanisms. Metal-based filaments [14,15], hydrogels [16,17], ceramics [18,19], and electrodes are being investigated for their ability to promote axonal growth, Schwann cell function, and overall functional recovery in damaged nerves. The unique properties of biofunctional metal ions, including their roles in neuroprotection [20], inflammation regulation [21], and stimulation of nerve growth [22,23], offer a new therapeutic direction in tissue engineering. Magnesium, for example, is valued for its neurotrophic factors [24] and anti-inflammatory effects [25], while zinc and lithium promote axonal regeneration [26] and reduce oxidative stress [27].

Researchers are working to enhance nerve regeneration by incorporating these ions into biodegradable and biocompatible materials, aiming to create an optimal environment for regeneration. In recent years, the benefits of electrical and magnetic stimulation in promoting neural regeneration have garnered significant attention [28]. These biophysical modalities can be effectively integrated into metal-based biomaterials, leveraging the intrinsic electrical conductivity of metals to modulate cellular behavior [29]. Advances in materials science have facilitated the development of long-lasting, biologically compatible metallic electrodes capable of chronic implantation within the nervous system, enabling localized stimulation and functional restoration [30]. Among the various candidates, iron (Fe), gold (Au), and silver (Ag) have emerged as exemplary materials, with multiple studies demonstrating their efficacy in supporting neural repair through combined electrical conductivity, bioactivity, and, in the case of Ag, antibacterial properties. However, challenges such as long-term biocompatibility [31], rapid degradation [32,33], mechanical properties, conductivity, and the need for specific optimization based on injury type must be addressed despite the potential of metal-based biomaterials.

These problems underscore how difficult it is to create nerve-healing materials that are both effective and safe while balancing controlled ion release with functional outcomes. This literature focuses on the contribution of biofunctional metals in promoting peripheral nerve regeneration, considering the most recent advancements in metal elements and the mechanisms that support nerve repair. Current issues and future directions of biodegradable metal-based materials for peripheral nerve regenerative medicine should be differentially addressed. This review also discusses the future of nerve guidance conduits (NGCs) and outlines prospective research illustrations. By identifying existing challenges and emerging opportunities, this work aims to inspire further exploration of innovative biomaterial-based strategies for peripheral nerve repair. By integrating interdisciplinary insights from biomaterials science, neurobiology, and tissue engineering, the review advances the frontiers of peripheral nerve regeneration and supports the development of clinically translatable solutions.

### Physiology of PNIs

Peripheral nerves contain several elements that enhance their functional capacity and structural integrity (Fig. 1A). This comprehensive review of these factors is crucial to understanding the pathophysiology of PNI and regeneration.

#### Structure of the peripheral nervous system

It is essential to consider that peripheral nerves are intricate structures composed of axons, Schwann cells, and connective tissue sheaths [34] (Fig. 1A). These components aid signal transmission and provide structural integrity to the nerve. To understand what happens after nerve injuries and during regeneration, it is crucial to clarify the functions of each component. The physiology of PNIs is complicated, necessitating a basic overview of these structures and their functions [35]. Myelin, a fatty sheath produced by Schwann cells, wraps around axons to prevent signal degradation over long distances. Schwann cells also assist with axonal debris clearance and generate bands of Büngner to guide regenerating axons toward their targets [36]. The connective tissue sheaths have an essential structural and protective role for the peripheral nerves. The innermost layer of tissue, the endoneurium, envelopes individual axons. At the same time, a group of these bundles into fascicles is encased in perineurium, and the nerve as a whole within epineurium imparts mechanical properties to withstand exogenous forces [37].

Schwann cells originate from neural crest cells and follow a developmental progression toward either a myelinating or nonmyelinating phenotype. Upon nerve injury, mature Schwann cells dedifferentiate into repair Schwann cells, which play a central role in axon guidance and regeneration (Fig. 1D). Following regeneration, these cells redifferentiate into either myelinating or nonmyelinating Schwann cells depending on axonal signals. The regeneration process of PNI includes sequential steps such as macrophage-mediated clearance of myelin debris, Schwann cell reprogramming, formation of bands of Büngner, axonal regrowth, and final remyelination (Fig. 1E).

#### Mechanisms of PNI and regeneration

PNIs result from trauma, compression, laceration, or disease, and their severity varies, dictating the potential for recovery. The Sunderland classification system categorizes these injuries, ranging from neurapraxia (mild) to neurotmesis (severe), which helps guide treatment approaches and predict outcomes [38] (Fig. 1B).

Neurapraxia, the least severe type, involves a temporary nerve conduction interruption without axonal damage, allowing for complete and rapid recovery [39]. In axonotmesis, there is damage to the axons, but the surrounding connective tissue sheaths, such as the endoneurium, perineurium, and epineurium, remain intact [40]. Wallerian degeneration occurs in this case, where the axon and myelin distal to the injury site degrade [41]. However, axonal regeneration is possible since the connective tissue framework is preserved. Neurotmesis, the most severe form of nerve injury, involves a complete disruption of the axons and the surrounding connective tissue sheaths [42]. Recovery in these cases is unlikely without surgical intervention, and even then, outcomes are often suboptimal. After an axonal injury, Wallerian degeneration begins. The distal axon and myelin degenerate in this process, and macrophages and Schwann cells work together to clear the debris. Macrophages remove myelin fragments and release signaling molecules that stimulate Schwann cell proliferation and their transition to a repair-supportive state. Schwann cells then align to form the bands of Büngner, creating pathways that guide regenerating axons toward their target tissues (Fig. 1C).

Biofunctional metal ions such as magnesium (Mg), zinc (Zn), and calcium (Ca) are essential for promoting nerve regeneration by modulating axonal and Schwann cell activities. These ions maintain the regenerative milieu by promoting cell proliferation, reducing inflammatory responses, and supporting the electrical conductance necessary for normal neuronal function [43]. For more severe injuries such as neurotmesis, when regenerative pathways are lost, or very limited in G0 phase tissues/organs, metal ion-based biomaterials provide a timely strategy to steer axonal regeneration for optimal functional outputs.

### Clinical challenge of PNIs

The limited ability for regeneration and the complexity of the architecture in peripheral nerves present significant clinical barriers to addressing injuries [44]. These injuries can lead to severe functional deficits, including impaired sensory perception, decreased voluntary motor function, and autonomic regulation, considerably diminishing patients’ quality of life. Symptoms may start mildly but progress to numbness, reduced pain sensitivity, and increased risk of damage. Injury to motor nerves results in impaired movement, muscle weakness, and atrophy. Autonomic nerve injuries complicate essential physiological functions, such as blood pressure regulation and bladder control, making daily life management more difficult.

This contributes to the slow reinnervation process, averaging 1 mm daily, prolonging recovery, and increasing the risk of denervation-related imbalances. Misdirected axon regeneration can hinder recovery, causing disordered sensations or movements that impair functional capacity. Interventions, from conventional nerve repair and grafting to innovative strategies, often result in suboptimal outcomes. Allografts risk immune rejection, and synthetic conduits may not support proper regeneration.

Rehabilitation is essential for recovery, requiring long-term physical and occupational therapy and sometimes electrical stimulation [45]. These treatments can be emotionally taxing for patients, causing depression or anxiety due to extended recovery and uncertain outcomes. Clinical results are influenced by factors like patient age, injury location, severity, and time before intervention, with many patients never fully regaining function, even with the best care. The financial burden of such injuries is substantial, covering medical care, rehabilitation, and lost productivity, impacting both individuals and the broader economy.

### Limitations of traditional treatments

Current traditional therapies for nerve injuries include surgical and nonsurgical methods, both of which have restrictions on enabling optimal nerve regeneration and functional recovery [5]. Despite decades of application, these methods fail to achieve complete nerve restoration. Surgical approaches include nerve grafts (autologous or allografts), nerve conduits, nerve transfer, fibrin glue, and cell-based therapies. Despite being the gold standard, autologous nerve grafts necessitate the removal of a patient’s nerve, which might result in secondary damage, chronic pain, and sensory loss at the donor site [46]. Allografts eliminate the need for self-harvesting but come with the risk of immune rejection, often requiring immunosuppressive treatments that can lead to infections [47]. Nerve conduits, designed to bridge nerve gaps with biocompatible materials, usually lack the bioactive properties needed for long-distance axonal growth, and their success is limited to significant nerve defects [48]. Nerve transfers [49] and fibrin glue [50] are helpful for small gaps but require precise surgical skills, and their outcomes are inconsistent. Cell-based therapies show potential in enhancing nerve repair, but their clinical application is still in the experimental stages, with issues surrounding cell survival and integration [51].

Nonsurgical approaches focus on less invasive strategies, including medications [52], phytochemicals [53], and electrical nerve stimulation [54]. While these methods aim to modulate the body’s natural healing processes, their effectiveness in achieving substantial nerve regeneration is limited. Medication does not directly stimulate nerve growth, although it can assist in controlling pain and inflammation. Phytochemicals derived from plants or herbs, mushrooms, and decoctions have demonstrated potential in preclinical studies [55]. Plant-derived compounds have given hope to scientists due to their pharmacological properties, including anti-inflammatory, anti-oxidative, and antimicrobial [5]. However, challenges remain regarding biocompatibility and consistency in clinical use applications. Electrical stimulation holds promise in supporting neuronal growth and functional rehabilitation after nerve injuries. Still, the efficacy of these devices is constrained by their reliance on particular stimulation parameters, limiting both portability and accessibility.

## Mechanism of Contributions of Biodegradable and Implantable Metals to Peripheral Nerve Regeneration

It is a prerequisite to understand peripheral nerve regeneration to treat through biofunctional metal ion-based regenerative engineering. Metal ions play a significant role in the regeneration of peripheral nerves by facilitating axonal growth and enhancing Schwann cell functions, encompassing proliferation, migration, and remyelination. Furthermore, these ions exert immunomodulatory influences by shifting macrophages from a pro-inflammatory (M1) phenotype toward an anti-inflammatory (M2) phenotype. Additionally, metal ions are utilized to develop electrically conductive biomaterials that foster neural regeneration by generating an electrical field. The mechanical characteristics of metal ions vary significantly, ranging from pliable materials such as ion-doped polymers, hydrogels, and solutions to rigid materials, including ceramics, particles, and filaments (Fig. 1C). It is essential to align the mechanical properties of these materials with those of the native neural tissue. Although these advancements are commendable, much more remains to be done to overcome the complex challenges of PNIs. This highlights the essential role of metal ions, such as magnesium, zinc, and calcium, in promoting nerve regeneration through their biofunctional properties, anti-inflammatory effects, and electrical conductivity. Incorporating these ions into biomaterials can significantly enhance axonal growth, Schwann cell regeneration, and overall nerve functional recovery [56]. Biofunctional metal ions represent a promising avenue for surgical and nonsurgical interventions, fostering a bioactive environment conducive to effective and sustained nerve regeneration. Their ability to address the limitations of conventional treatments highlights the importance of continued research and development in this area.

### Axonal regeneration and functional recovery

#### Axonal growth guidance modulation

**Fig. 2.**
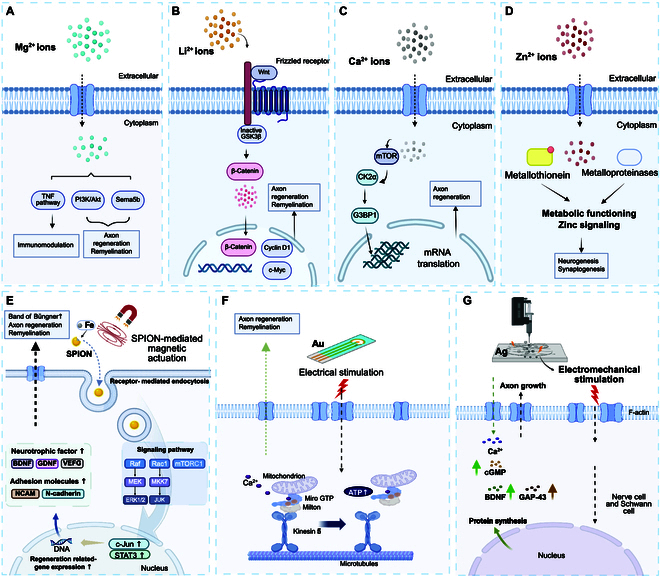
Illustration of molecular mechanisms of metal-based peripheral nerve regeneration. (A) Mg^2+^ enters cells via transporters, activating key pathways such as TNF and PI3K/Akt, and stimulating Sema5b to support immunomodulation, axon regeneration, and remyelination. (B) Li^+^ activates the Wnt/β-catenin signaling pathway, where the frizzled receptor inactivates GSK3β, enabling β-catenin to up-regulate c-Myc and cyclin D1 for facilitating axon regeneration and remyelination. (C) Role of calcium ions (Ca^2+^) in axon regeneration through mTOR pathway. (D) Role of zinc ions (Zn^2+^) in synaptic transmission and neurogenesis. Illustration of iron and gold ion-based promotes nerve regeneration. (E) Schematic illustration of the mechanism by which SPION-mediated magnetic actuation promotes peripheral nerve regeneration. SPIONs exert mechanical stimulation inside the cell, which activates intracellular signaling pathways promoting the formation of bands of Büngner, axon regeneration, and remyelination, which are critical for peripheral nerve repair. (F) Gold (Au)-based electrodes provide electrical stimulation, enhancing axon growth by boosting mitochondrial transport via Kinesin 5, Miro GTP, and Milton, which increases ATP production to support neuronal repair [64]. (G) Ag-based electrodes, when subjected to electrical stimulation, up-regulate the secretion of growth factors such as FGF, CNTF, and GDNF, while electromechanical stimulation enhances axon growth and cell proliferation.

Axonal regeneration is a central process of peripheral nerve regeneration [57]. When peripheral nerves are damaged, the regrowth of axons is a critical step in reestablishing these connections, without which full neurological recovery is impossible. According to Yao et al. [58], magnesium ions (Mg^2+^) promote axonal growth during peripheral nerve regeneration by activating the phosphatidylinositol 3-kinase (PI3K)/Akt signaling pathway and up-regulating axon guidance molecules like Sema5b​ (Fig. 2A). Their study demonstrated that Mg^2+^ ion, when incorporated into a bisphosphonate-based injectable hydrogel, enhances neurite outgrowth in a concentration-dependent manner. In vivo experiments using polycaprolactone (PCL) conduits filled with Mg^2+^ ion-releasing hydrogel confirmed significant axonal regeneration and functional recovery in rats with sciatic nerve injuries. This suggests that the controlled release of Mg^2+^ is critical to facilitating axonal regrowth and nerve repair. According to Kocman et al.​ [59], lithium ions (Li^+^) facilitate axonal growth in peripheral nerve regeneration by inhibiting the glycogen synthase kinase 3β (GSK3β) enzyme, which activates the Wnt/β-catenin signaling pathway (Fig. 2B). This process promotes axonal outgrowth and myelination, essential for nerve repair. In their study, lithium-loaded hyaluronic acid hydrogels were applied to injured sciatic nerves in rats, demonstrating improved axonal regeneration and motor function recovery. The controlled release of Li^+^ from the hydrogel provides localized and sustained stimulation for axonal growth. These findings suggest that lithium can effectively enhance nerve regeneration through molecular signaling modulation. Furthermore, during nerve injury, elevated Ca^2+^ levels drive specificity by altering axoplasmic Ca^2+^ ions, sequentially triggering axonal synthesis of mechanistic target of rapamycin (mTOR) and casein kinase 2α (CK2α). Axonal G3BP1 granule disassembly, triggered by CK2α phosphorylation at Ser^149^, releases mRNAs for local translation, thereby accelerating axon regeneration (Fig. 2C) [60].

#### Schwann cell proliferation and differentiation

Schwann cells are central to peripheral nerve regeneration due to their multifaceted role in axonal support and myelination [61]. Upon nerve injury, Schwann cells dedifferentiate, proliferate, and migrate to the injury site, where they facilitate the repair process by forming bands of Büngner—structures that guide the regeneration of axons [62,63]. Therefore, efficient regeneration of Schwann cells is critical for restoring nerve function. In the context of Schwann cell regeneration, metal ions contribute significantly to regulating cellular processes that govern cell proliferation, migration, and myelination [64]. Zinc-dependent enzymes, such as metalloproteinases, and zinc-binding proteins, such as metallothioneins, have a significant function in zinc metabolism and signaling pathways. Additionally, metallothioneins maintain the homeostasis of essential trace elements and heavy metal detoxification and scavenge reactive oxygen species (ROS), while zinc influences synaptic transmission, affecting neuronal communication, synaptic plasticity, and Schwann cell myelination (Fig. 2D) [65]. One key mechanism involves modulating intracellular signaling pathways that control Schwann cell behavior in response to nerve damage. According to Qian et al. [66], a piezoelectric zinc oxide (ZnO) nanogenerator scaffold is manufactured. Its biocompatibility supplies Schwann cells with adhesion and proliferation, as reflected by the higher expression of nerve growth factor (NGF) and vascular endothelial growth factor (VEGF). Stratton et al. [67] developed a biofunctional scaffold consisting of aligned fibers of PCL with embedded ZnO nanoparticles. Incorporating metal ions and ZnOs within PCL improved Schwann cell growth and Schwann cell-mediated axon extension. Additionally, the piezoelectric effect of ZnO generates localized electrical stimulation, promoting axon remyelination. This mechanism enhances nerve regeneration and functional recovery after PNI. Liu et al. [30] report that by magnetic actuation, superparamagnetic iron oxide nanoparticles (SPIONs) stimulate Schwann cell regeneration. This mechanism induces and stabilizes repair-supportive phenotypes in Schwann cells, which are essential for nerve regeneration. SPIONs, under a magnetic field, generate mechanical forces that Schwann cells transduce into intracellular biochemical signals, enhancing their repair function (Fig. 2E). This process activates Schwann cell elongation. It creates a favorable microenvironment for the extension and growth of new regenerative axons. The regulation of metal ions in reconstructing damaged Schwann cells is related to growth factors, energy metabolism, and intracellular signaling pathways within a specific range. These metal ions are crucial for Schwann cell proliferation, migration, and myelination, all of which are necessary for successful nerve regeneration.

#### Immunomodulation via macrophage polarization

Recent studies have highlighted the critical role of macrophages in peripheral nerve regeneration [68]. Macrophages can polarize into a pro-inflammatory (M1 phenotype) or an anti-inflammatory, tissue-repairing (M2 phenotype) state. Magnesium promotes polarization toward the M2 phenotype, increasing anti-inflammatory cytokine production and reducing pro-inflammatory ones [25]. This shift helps prevent chronic inflammation, protects Schwann cells from apoptosis, and supports motor neuron survival after sciatic nerve injury. Magnesium regulates this process by transitioning macrophages from the inflammatory M1 to the anti-inflammatory M2 phenotype, creating a regenerative environment [69]. For instance, magnesium supplementation significantly reduces pro-inflammatory cytokines like tumor necrosis factor-α (TNF-α), interleukin-1β (IL-1β), IL-6, and interferon-γ (IFN-γ), thereby facilitating nerve regeneration. Additionally, magnesium decreases the release of MCP-1, a critical factor in inflammation, preventing chronic inflammation. Magnesium–lithium alloys (Mg–1.6 wt % Li) reduce inflammation in damaged nerve environments, enhancing the anti-inflammatory effects of magnesium [70]. Similarly, Li–Mg–Si (LMS) bioceramics polarize macrophages toward the M2 phenotype, further inhibiting inflammation and supporting tissue regeneration [18]. A study by Foroutan Koudehi et al. [71] found that incorporating silver nanoparticles (AgNPs) into a nano-bioglass/gelatin scaffold provides antimicrobial and immunomodulatory effects essential for peripheral nerve regeneration. The anti-infective potential of AgNPs helps reduce infection risk and orchestrates an appropriate immune response, preventing excessive inflammation. This creates a more favorable environment for nerve healing, as inflammation can slow the process. Additionally, the anti-inflammatory effects of silver nanoparticles promote better tissue healing and axon regrowth. With the rapid development of material engineering, metal ions have emerged as a promising pathway to promote peripheral nerve regeneration. They can modulate macrophage polarization, decrease pro-inflammatory cytokines, and create an environment conducive to tissue regeneration, making them an attractive area for further research and potential clinical applications.

### Electrical conductivity and electrostimulation

Electrical conductivity plays a vital role in peripheral nerve regeneration by enhancing the bioactivity of materials used in nerve repair and mimicking the natural electrical signals of the nervous system [72,73]. For nerve regeneration to be successful, conductive materials must drive biological processes, including neuron proliferation, differentiation, and axonal guidance [74]. These materials can facilitate the transmission of electrical signals between damaged neurons, promoting functional recovery. According to Choi et al. [75], magnesium-based bioresorbable electronic stimulators enhance peripheral nerve regeneration through electrical conductivity​. These devices use dynamic covalent polymers to provide wounded nerves with continuous electrical stimulation, encouraging axonal development and enhancing functional recovery. The wireless, battery-free system ensures long-term electrical stimulation, facilitating neuromuscular regeneration by maintaining nerve electrical continuity. Au ions facilitate the transmission of electrical signals between neurons by mimicking the natural bioelectrical environment, thus promoting neurite outgrowth and improving the differentiation of neural cells. Notably, Bai et al. [76] showed that SNGC-WES (self-curling silk NGC incorporating a wireless electrical stimulator) successfully bridged a 10-mm gap in a rat sciatic nerve defect model, facilitating axonal regeneration and functional recovery comparable to autografts. It provided stable and effective noninvasive electrical stimulation, with favorable biodegradation noted within 7 d. Histological analyses showed organized regenerated axons and Schwann cells, with improved myelination and axon diameter in the SNGC-WES group. Electrophysiological evaluations indicated enhanced nerve conduction velocity and compound muscle action potential over time (Fig. 2F). Key signaling pathways related to mitochondrial movement [adenosine triphosphate (ATP) supply], particularly calcium and metabolic pathways, were up-regulated in the SNGC-WES group, which is crucial for axon regeneration. RNA sequencing revealed 773 up-regulated and 460 down-regulated differentially expressed genes, highlighting the involvement of various genes in the regeneration process. Silver-based materials have demonstrated potential for nerve regeneration due to their antibacterial activity and electrical conductivity. When subjected to electrical stimulation, they up-regulate the secretion of growth factors such as fibroblast growth factor (FGF), ciliary neurotrophic factor (CNTF), and glial cell line-derived neurotrophic factor (GDNF), promoting axon growth. Electromechanical stimulation also supports axon growth and cell proliferation. Optimizing electrical conductivity ensures that conductive scaffolds effectively facilitate nerve signal transmission and support regeneration.

### Mechanical compatibility and structural integration

**Fig. 3.**
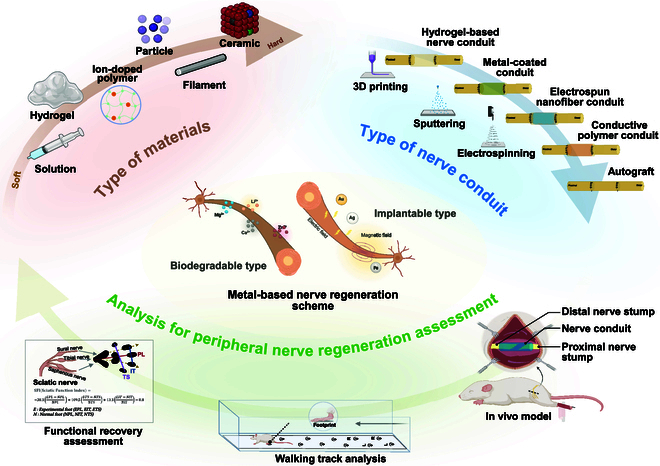
Overview of metal-based strategies and evaluation methods for peripheral nerve regeneration. The schematic illustrates an integrated perspective on material types, nerve conduit design, and assessment techniques used in metal-based peripheral nerve regeneration research. At the center, metal-based nerve regeneration mechanisms are categorized into biodegradable and implantable systems. Biodegradable metals (e.g., Mg^2+^, Zn^2+^, Ca^2+^, and Li^+^) release bioactive ions that influence regeneration pathways. Implantable metals (e.g., Au, Ag, and Fe) may interact with electric or magnetic fields to stimulate axonal growth and inflammation modulation. At the top left (brown), biomaterials are classified by physical state and hardness, ranging from soft solutions and hydrogels to ion-doped polymers, particles, filaments, and ceramics. At the top right (blue) are various fabrication methods and types of NGCs, including hydrogel-based conduits, metal-coated scaffolds, electrospun nanofibers, conductive polymer conduits, and autografts. Fabrication techniques like 3D printing, sputtering, and electrospinning enable controlled architecture and functional integration. Finally, at the bottom, an in vivo experimental setup showing nerve conduit implantation bridging proximal and distal nerve stumps in a rodent sciatic nerve injury model. Methods for functional recovery assessment, including walking track analysis and calculation of the SFI based on hindlimb footprint parameters and sciatic nerve distribution.

Mechanical compatibility is crucial in peripheral nerve regeneration [77,78]. It ensures that the materials used for nerve repair can physically support the regenerating tissue without causing additional damage or stress [79]. For nerve regeneration to be successful, the materials used must closely resemble the softness and flexibility of the original nerve tissue to create the ideal environment for the growth and regeneration of nerve cells [80]. If the material is too stiff or rigid, it can mechanically mismatch with surrounding tissue, leading to inflammation, scarring, or poor nerve healing [81]. However, softer materials may not offer sufficient stiffness for regenerating axons to push against [82]. This balance between strength and flexibility enables scaffolds or conduits to remain stable under implantation conditions while promoting cell migration and axonal extension. Similarly, mechanical compatibility is required to encourage tissue nerve regeneration by decreasing mechanical strain on regenerating fibers and providing structural support for growth. Aydemir Sezer et al. [83] found that zero-valent iron (Fe) nanoparticles improve mechanical compatibility in peripheral nerve regeneration by increasing the tensile properties of PCL nanofibers. These iron-loaded biomaterials increase their strength and flexibility, making them ideal for soft tissue applications such as nerve restoration. The scaffold’s mechanical compatibility ensures better suture handling and implant stability, supporting effective peripheral nerve regeneration (Fig. 3).

## Metal-Based Biomaterials for Peripheral Nerve Regeneration

### Biodegradable metal

Metal ions are not typically used in their free form in peripheral nerves as they can quickly spread through the bloodstream and negatively affect multiple organs [84,85]. To address this issue, researchers often incorporate metal ions into biomaterials to impact specific release kinetics, optimize their ability, and minimize adverse effects on healthy tissue [86]. Metal ion integration into biomaterials can increase biofunctionality and reduce harmful effects. Different metal ion-based materials have recently been developed to treat PNIs. This review discussed metal ion-based materials with advances achieved in recent years through tissue engineering and regenerative medicine research.

#### Magnesium ion

It was previously shown that magnesium metal is a suitable approach for peripheral nerve repair. However, the studies are still relatively immature and offer initial insights into its potential to improve nervous function [14]. The study used pure magnesium metal filaments in a rat sciatic nerve injury model (Fig. 3). These filaments displayed good biocompatibility and lower local inflammation and facilitated muscle mass recovery, making them exciting candidates for developing an ideal environment for nerve regeneration. Nevertheless, the fast corrosion rate of pure magnesium and hydrogen gas generation must be addressed [87]. Therefore, additional treatments, such as protective coatings or alloying with other elements, must be applied to enhance its stability [88]. A family of materials based on magnesium called metal-based glasses is a heterogeneous material with the beneficial properties of both glass and metal [89]. Due to their amorphous (noncrystalline) structure, they exhibit high strength and enhanced corrosion resistance. Monfared et al. [90] reported a layer-by-layer coating of a tannic acid (TA)/poly(N-vinylpyrrolidone) to Mg-based glass for nerve tissue regeneration. The coating improved corrosion resistance and biocompatibility, promoting Schwann cell viability. Magnesium-doped biomaterials combine magnesium with other bioactive materials to leverage each component’s strengths. These composites aim to optimize degradation rates, mechanical properties, and biological activity, providing a synergistic approach to nerve regeneration. Gao et al. [16] reported advancing neural regeneration via adaptable hydrogels enriched with Mg^2+^ and silk fibroin (SF). This study demonstrated that hydrogels enriched with Mg^2+^ and SF promoted cell infiltration and macrophage polarization. These hydrogels significantly aided nerve regeneration by providing structural support and enhancing the bioactivity of the scaffold, creating an optimal environment for nerve repair. In another study, Cai et al. [9] developed the GDNF-Gel/HA-Mg conduit, a magnesium-based nerve repair device designed to address the limitations of traditional magnesium conduits, such as poor mechanical support and rapid degradation. This conduit features a hydroxyapatite (HA) coating (Fig. 3) for improved corrosion resistance and a gelatin methacryloyl (GelMA) hydrogel for sustained release of GDNF, vital for nerve regeneration. Quantitative analysis showed that GDNF-Gel/HA-Mg enhances Schwann cell maturity and dedifferentiation, indicated by increased expression of key markers at 12 weeks postoperatively. These changes are essential for Schwann cell migration, axonal regrowth, and remyelination, promoting effective nerve repair and recovery. Furthermore, Sun et al. [18] showed LMS bioceramics for peripheral nerve regeneration where treatment modulated immune responses, promoted Schwann cell functions, and enhanced nerve regeneration and functional recovery. These bioceramics created a dynamic immunomodulatory and repair-supportive microenvironment, leveraging the bioactivity of Mg^2+^ and the structural benefits of ceramics to support nerve repair. Advances in Mg^2+^-based nerve conduits highlight the versatility and potential of magnesium in nerve regeneration. Pure magnesium offers significant benefits but is challenged by rapid degradation. Magnesium alloys present a solution with improved mechanical properties and slower degradation rates, while magnesium-based glasses provide high strength and corrosion resistance [91]. Surface modifications improve the performance and applicability of magnesium-based materials for biomedical uses. Magnesium-doped biomaterials combine the benefits of different materials, creating synergistic effects that enhance nerve repair. Further research and development are needed to fully harness the clinical potential of Mg^2+^ in biomaterials for peripheral nerve regeneration and improve patient outcomes and quality of life.

#### Lithium ion

Lithium can positively regulate the myelination process through nerve cell proliferation. In the last 15 years, many groups have reported the lithium-based treatment of different diseases related to the nervous system. Likewise, lithium ion-based biomaterials also ameliorated PNIs. Lithium exerts nerve regeneration through the Wnt/β-catenin signaling pathway. Increased lithium ions can up-regulate Wnt protein, and its binding to surface receptors is called frizzled protein, which initiates the Wnt downstream pathways. The frizzled protein inactivates GSK3β enzymes and activates β-catenin to accumulate in the cytoplasm. The β-catenin protein enters the nucleus and up-regulates cells to produce c-myc and cyclin D1. Cyclin D1 protein up-regulation is highly linked with Schwann cell proliferation [92], whereas c-myc correlates with Schwann cell repair and regeneration [93]. These mechanistic studies increased the understanding of material scientists to work on developing lithium ion-based peripheral nerve regeneration schemes. The study by Gu et al. [94] found that lithium chloride (LiCl) increases Schwann cell proliferation and blocks migration. LiCl, at 5 to 30 mM concentrations, enhanced Schwann cell viability and proliferation in a dose-dependent manner but significantly reduced chemoattractant-induced migration at higher dosages. These findings suggest that lithium is a promising therapeutic candidate for peripheral nerve repair. Dolatyar et al. [13] revealed that local delivery of Li^+^ from electrospun nanofibrous PLA (polylactide) could promote peripheral nerve regeneration in vivo. These scaffolds allow controlled and sustained Li^+^ release, activating the Wnt/β-catenin signaling pathway by inducing Schwann cell differentiation and axonal growth. Combining lithium ions (Li^+^) with biomaterials like nano-HA greatly favors peripheral nerve regeneration by enhancing neurite outgrowth and mitochondrial function. Li^+^-doped nHAp also reduces oxidative stress and nitric oxide levels, which are crucial for nerve protection and recovery [95]. Recently, Yang et al. [96] reported that lithium-carbonized polymer dots (Li-CPDs) significantly enhanced the removal of myelin debris and promoted nerve regeneration by activating autophagy in Schwann cells after PNI. Histological evaluations showed that Li-CPDs effectively reduced degenerated myelin and replaced it with newly formed myelin sheaths. Functional recovery assessments indicated that the sciatic function index (SFI) values were higher in the Li-CPDs group compared to the control group. Additionally, Li-CPDs exhibited minimal renal toxicity, suggesting improved biosafety over traditional lithium drugs. This approach permits regional and gradual lithium release, reducing toxicity while supporting nerve regeneration.

#### Calcium ion

The biological functions of calcium-based biomaterials extend beyond bone mineralization and have emerged as potential tools for peripheral nerve repair [97,98]. While most of the body’s calcium is stored in bones and teeth, calcium ions are crucial in neuronal signaling, affecting growth cone behavior and axonal restoration during nerve outgrowth and repair. This has led to growing interest in using calcium-containing biomaterials for nerve tissue engineering (Table 1). Based on Sahoo et al. [60], Ca^2+^ plays a crucial role in peripheral nerve regeneration by enabling local protein synthesis within axons. Ca^2+^ is also incorporated into biomaterials, such as calcium-dependent hydrogel systems, to control local intracellular signaling pathways essential for stimulating axonal growth. These cascades enable Ca^2+^ to regulate the phosphorylation of essential proteins, like CK2α, involved in disassembling mRNA–protein granules that impede nerve regrowth, thus enhancing repair. Bu et al. [99] present the conductive sodium alginate (SA) and carboxymethyl chitosan (CMCS) polymer hydrogels (SA/CMCS/PPy), which can release sustained calcium ions. The advantages of conductive hydrogels demonstrate controlling electrical stimulation, good biocompatibility, and nerve regeneration (Table 1). Hybrid scaffolds provide Ca^2+^ for the functional activity of bioactive materials like porous chitosan/CaTiO₃ scaffolds, which play a promising role in sciatic nerve regeneration [100]​. Since Schwann cells are crucial for peripheral nerve repair, these scaffolds enhance Schwann cell attachment and proliferation, creating a supportive environment for their biological function (Table 1). Furthermore, Ca^2+^ regulates cell signaling pathways, promoting cellular protrusion and the secretion of neurotrophins like NGF, CNTF, and BDNF (brain-derived neurotrophic factor), which are essential for nerve regeneration. Nawrotek et al. [101] developed osteoconductive Ca^2+^-releasing chitosan-based hydrogel implants designed to guide peripheral nerve regeneration by promoting Schwann cell activity and axonal growth while enhancing mechanical strength. The controlled release of Ca^2+^ is critical for regulating intracellular signaling cascades necessary for nerve repair. Additionally, the biocompatible nature of these biomaterials supports a mild inflammatory response at the injury site, creating an optimal environment for successful nerve regeneration.

**Table 1.** Metal-based regenerative strategies for peripheral nerve injury

Metal ion	Material type	Experiment type	Mechanism	Key findings	Year
Mg	Magnesium filament	In vivo/Lewis rat	Anti-inflammation Axonal growth Controlled ion release	Biodegradable and promotes nerve regeneration. It degrades faster in acidic environments without hindering the regeneration processes.	2015 [14]
	Magnesium wire	In vivo/Sprague–Dawley (SD) rat	Anti-inflammation Schwann cell nerve growth, secretion, and myelination Axonal growth Modulation of signaling pathway	Exhibits anti-inflammatory properties, promotes Schwann cell activation, and enhances the secretion of nerve growth factors, facilitating axon regeneration and myelination, leading to the recovery of nerve function.	2016 [24]
	Magnesium filament	In vivo/Lewis rat	Anti-inflammation Axonal growth Controlled ion release	Biodegradable material that supports axonal regeneration in short nerve gap injuries, promoting nerve regeneration while minimizing scar tissue formation.	2017 [146]
	Electrode	In vitro/L-929 In vivo/CD1 mice	Neuroprotection Axonal growth Controlled ion release Electrical conductivity	Effectively promote nerve injury recovery and maintain muscle function through electrical stimulation. They are considered a safe therapeutic platform that naturally degrades without requiring additional surgery.	2020 [75]
	S/Mg-SF/CS nerve guidance conduit (NGC)	In vitro/RSC-96 In vivo/Sprague–Dawley (SD) rat	Schwann cell myelination Axonal growth controlled ion release	Provides physical stability and biocompatibility with Schwann cells, promoting axon growth and myelination and aiding in nerve regeneration.	2021 [147]
	Mg-encapsulated hydrogel	In vitro/primary dorsal root ganglion (DRG) neuron In vivo/Sprague–Dawley (SD) rat	Neuroprotection Schwann cell myelination Axonal growth Modulation of signaling pathway Controlled ion release	Fundamental mechanisms include axon regeneration via Mg^2+^ release, the PI3K/Akt signaling pathway, and Schwann cell myelination.	2022 [58]
	Electrode	In vivo/CD1 mice, Lewis rat	Neuroprotection Controlled ion release Electrical conductivity	It effectively manages pain by blocking electrical signals and has the potential to decrease the reliance on addictive drugs like opioids by acting as an alternative.	2022 [143]
	Li–Mg–Si ceramic	In vitro/RSC96, RAW264.7 In vivo/Sprague–Dawley (SD) rat	Anti-inflammation Schwann cell migration and differentiation, myelination Axonal growth Modulation of signaling pathway	Promotes M2 macrophage polarization and Schwann cell myelination, supporting immune microenvironment regulation and nerve regeneration.	2023 [18]
	Magnesium-releasing hydrogel	In vitro/primary dorsal root ganglion (DRG) neuron, primary Schwann cell, primary fibroblast cell, RAW264.7 In vivo/Sprague–Dawley (SD) rat	Anti-inflammation Schwann cell migration Axon growth Controlled ion release	It promotes Schwann cell and macrophage infiltration, encourages axonal growth, and induces macrophage polarization to create a supportive microenvironment for nerve regeneration and functional recovery.	2024 [16]
Li	Lithium chloride injection	In vivo/Lewis rat	Schwann cell proliferation Axonal growth	When combined with GDNF, it facilitated Schwann cell activation and axon regeneration, enhancing nerve regeneration and fiber density in a 15-mm nerve defect model	2013 [148]
	Lithium chloride	In vivo/Sprague–Dawley (SD) rat	Axonal growth Modulation of signaling pathway	Promoted axon regeneration through GSK3β inhibition and increased axon elongation in damaged motor neurons	2014 [149]
	Lithium chloride	In vitro/primary Schwann cell	Schwann cell proliferation, myelination	Stimulated Schwann cell proliferation while inhibiting cell migration, influencing the specific role of Schwann cells in peripheral nerve regeneration	2020 [94]
	Lithium chloride	In vivo/Sprague–Dawley (SD) rat	Neuroprotection Anti-inflammation Axonal growth Modulation of signaling pathway Controlled ion release	Demonstrated neuroprotective and anti-inflammatory effects through GSK3β inhibition, with 5 mEq LiCl being the most effective for nerve regeneration	2020 [59]
	Lithium-containing octa-PEG adhesive	In vitro/primary Schwann cell In vivo/Sprague–Dawley (SD) rat	Anti-inflammation Axonal growth Controlled ion release	Accelerated axon regeneration and nerve reconnection more rapidly than traditional suture methods while reducing inflammation and fibrosis to enhance functional recovery	2020 [131]
	Li^+^ and Eu^3+^ ion-doped nanohydroxyapatite (nHAp)	In vitro/SH-SY5Y, PC12	Neuroprotection Axonal growth Controlled ion release	Exhibited antioxidant properties and promoted nerve regeneration by increasing neurite length	2021 [95]
	Li-doped polymer	In vitro/RSC96 In vivo/Sprague–Dawley (SD) rat	Activate Schwan cell autophagy to remove myelin debris Axonal growth Modulation of signaling pathway Controlled ion release	Activated autophagy in Schwann cells, promoting nerve regeneration and significantly reducing kidney toxicity, thus improving the safety of lithium-based treatments	2023 [96]
	LMS ceramic	In vitro/RSC96, RAW264.7 In vivo/Sprague–Dawley (SD) rat	Anti-inflammation Schwann cell migration, differentiation, myelination Modulation of signaling pathway	Promoted M2 macrophage polarization and Schwann cell myelination, supporting nerve regeneration and functional recovery by modulating the immune microenvironment	2023 [18]
	Lithium-loaded scaffold	In vitro/primary human adipose-derived mesenchymal stem cell In vivo/Wistar rat	Schwann cell differentiation, myelination Axonal growth Modulation of signaling pathway Controlled ion release	Promoted the differentiation of hADMSCs into Schwann cells and enhanced axon regeneration, contributing to nerve function recovery through activation of the Wnt/β-catenin pathway	2024 [13]
Ca	Calcium alginate	In vivo/Sprague–Dawley (SD) rat	Continuous release of BDNF Promotion of axonal growth	BDNF was continuously delivered via a calcium alginate release system, promoting axon regeneration and significantly reducing neuropathic pain	2006 [150]
	Calcium (Ca^2+^) absorption	In vivo/Sprague–Dawley (SD) rat	Axonal growth Controlled ion release	The recovery after nerve injury is strongly associated with a significant correlation between calcium absorption and the recovery of compound muscle action potential (CMAP)	2010 [151]
Ca	Nifedipine and calcitonin	In vivo/Sprague–Dawley (SD) rat	Schwann cell Axonal growth Controlled ion release	The promotion of calcium absorption by using nifedipine and calcitonin enhanced nerve regeneration and significantly improved nerve function recovery	2013 [97]
	Calcium alginate	In vivo/Wistar rat	Anti-inflammation Schwann cell activity Axonal growth Controlled ion release	Facilitated axon regeneration through interactions with inflammatory cells, supporting nerve regeneration at a level comparable to nerve autografts	2013 [98]
	Chitosan-hydroxyapatite-based hydrogel	In vitro/L929, THP-1	Anti-inflammation Controlled ion release	Demonstrated biocompatibility and showed potential for nerve regeneration through the release of calcium ions	2016 [101]
	NT-3 released from CaP-coated sutures	In vitro/NT-3	Axonal growth Controlled ion release	Promoted axon regeneration, and PNG (peripheral nerve graft) effectively acted as a support structure for nerve regeneration	2016 [152]
	CaTiO_3_ nanoparticle-containing chitosan (CS) hybrid scaffold	In vitro/primary Schwann cell	Schwann cell adhesion, proliferation Controlled ion release	Showed potential for contributing to peripheral nerve regeneration by promoting Schwann cell attachment and proliferation	2017 [100]
	Conductive SA/CMCS/PPy hydrogel	In vitro/PC12, RSC96, BMMSC In vivo/Sprague–Dawley (SD) rat	Schwann cell activity Axonal growth Controlled ion release Electrical conductivity Anti-inflammation	Conductive SA/CMCS/PPy showed good biocompatibility and repair features as a bioactive biomaterial.	2018 [99]
	PGS/CaTiO_₃_ nanocomposite	In vitro/PC12	Controlled ion release Axonal growth	The PGS/CaTiO_₃_ nanoparticle nanocomposite showed axonal outgrowth and extension with desirable effects on peripheral nerve regeneration.	2018 [153]
Zn	Zinc-silicate bioactive glass	In vitro/L929	Controlled ion release	Significantly impacted the biocompatibility and tensile strength of nerve guidance conduits (NGCs), providing an optimal material composition for cell viability and mechanical properties	2011 [154]
	Bioactive borate glass (BBG) doped with iron, gallium, and zinc	In vitro/primary dorsal root ganglion (DRG) neuron	Neuroprotection Axonal growth Controlled ion release	It enhances neuron survival and neurite growth, making it a promising material for peripheral nerve regeneration.	2016 [89]
	Piezoelectric zinc oxide nanogenerator scaffold	In vitro/RSC96 In vivo/Sprague–Dawley (SD) rat	Schwann cell adhesion, proliferation, myelination Axonal growth Modulation of signaling pathway Electrical conductivity	Provides an electrically conductive microenvironment through mechanical stimulation, accelerating Schwann cell proliferation and axonal regeneration, thereby speeding up peripheral nerve regeneration.	2020 [66]
	Zinc chloride/polycaprolactone conduit	In vitro/primary MSC	Anti-inflammation Axonal growth Controlled ion release	Exhibits antibacterial properties and promotes stem cell growth, potentially contributing to peripheral nerve regeneration.	2021 [65]
	Zn–2% Fe alloy filament	In vitro/primary Schwann cell, primary dorsal root ganglia (DRG) neuron	Anti-inflammation Axonal growth Controlled ion release	Exhibits biodegradability and contributes to axon density recovery and nerve regeneration, demonstrating biocompatibility without triggering an inflammatory response.	2023 [15]
Zn	Bionic microneedle NGCs (MNGCs)	In vitro/STC30007G-1 In vivo/Sprague–Dawley (SD) rat	Schwann cell activity Electrical conductivity Axon growth	Self-powered MNGCs demonstrated the capability to generate bioelectrical stimulation and promote axonal growth, myelination, neovascularization, and functional recovery of regenerative nerves.	2023 [15]
	Zinc-molybdenum-based biodegradable batteries	In vitro/primary Schwann cell In vivo/Sprague–Dawley (SD) rat	Schwann cell activity Axonal growth Electrical conductivity	Promote Schwann cell proliferation and nerve regeneration through electrical stimulation, improving motor function recovery in a 10-mm nerve injury model.	2024 [116]
Fe	Conjugated neurotrophic factors	In vitro/primary dorsal root ganglion (DRG) neuron	Neuroprotection Schwann cell myelination Axonal growth Modulation of signaling pathway Controlled ion release	Conjugated neurotrophic factors, especially conjugated GDNF, rapidly promoted nerve fiber sprouting and myelination, providing long-term and stable effects for nerve regeneration	2014 [155]
	Iron sulfate	In vivo/frog (*Rana esculenta*)	Neuroprotection Schwann cell proliferation Axonal growth Controlled ion release	Positively impacted nerve regeneration by increasing Schwann cell numbers and preventing myelinated axon degeneration	2017 [107]
	Magnetically templated hydrogels	In vitro/primary dorsal root ganglion (DRG) neuron In vivo/Lewis rat	Axonal growth Controlled ion release	Facilitated axon extension and nerve regeneration through aligned tubular microstructures, improving nerve recovery in a 10-mm nerve defect model	2020 [156]
	PCL/Fe10 nanofibers containing zero-valent iron (Fe) nanoparticles	In vitro/NIH/3T3, U87, SH-SY5Y, primary dorsal root ganglion (DRG) neuron	Axonal growth Controlled ion release Electrical conductivity	Enhanced electrical conductivity and mechanical properties, promoting axon growth and positively influencing nerve regeneration	2020 [83]
	SPION-mediated magnetic targeting of AdMSC	In vitro/primary AdMSC In vivo/Wistar rat	Neuroprotection Schwann cell Axonal growth Electrical conductivity	Directed AdMSCs to the injured nerve site, promoting nerve regeneration and myelination, which enhanced nerve conduction and functional recovery	2021 [157]
	CMNPs (nanoparticle)	In vivo/Wistar male	Neuroprotection Schwann cell Axonal growth Modulation of signaling pathway Controlled ion release	Promoted peripheral nerve injury recovery, positively impacting nerve function restoration and pain reduction. They are suggested as a promising treatment for nerve regeneration through increased NGF levels and axon regeneration	2021 [158]
	SPION	In vitro/RSC96 In vivo/Sprague–Dawley (SD) rat	Schwann cell Axonal growth modulation of signaling pathway Electrical conductivity	Maintained Schwann cells in a repair-supportive state, promoting nerve regeneration and functional recovery, suggesting a novel strategy for peripheral nerve injury treatment	2022 [30]
	2% IONPs/fibrin hydrogel	In vitro/PC12 In vivo/Sprague–Dawley (SD) rat	Anti-inflammation Axonal growth Modulation of signaling pathway	Increased the expression of NGF and VEGF, promoting nerve regeneration and improving motor function recovery to the level of autografts in a 10-mm nerve defect model	2024 [108]
Au	Chitosan–Au nanocomposite	In vitro/BCRC-60046, primary NSC In vivo/Sprague–Dawley (SD) rats	Neuroprotection Axonal growth Electrical conductivity	Provided mechanical strength, enhanced the performance of neural stem cells (NSCs) and glial cells, and contributed to nerve regeneration. The microstructured conduit promoted axonal regeneration and angiogenesis, making it a promising peripheral nerve injury repair alternative.	2008 [159]
	Au-PLLA scaffold	In vitro/NG108-15	Electrical conductivity	Offered electrical conductivity and mechanical strength, promoting muscle cell elongation and fusion, showing potential biocompatibility for muscle regeneration.	2011 [160]
	Au nanorods with 780-nm laser irradiation	In vivo/Sprague–Dawley (SD) rats	Axonal growth	Stimulated neurite growth offers new possibilities for peripheral nerve regeneration and treatment of nervous system injuries.	2013 [161]
	Silk–gold nanocomposite nerve conduits	In vitro/Schwann cell, PC12	Neuroprotection Anti-inflammation Schwann cell myelination Axonal growth Electrical conductivity	Preseeded with Schwann cells, these conduits promoted myelination and axon regeneration, significantly improving nerve conduction velocity and muscle function and demonstrating excellent performance in nerve injury recovery.	2015 [162]
	Graphene-decorated AuNPs and P3HB-co-HHx conductive nanofiber scaffold	In vitro/PC12	Schwann cell proliferation, migration Controlled ion release Electrical conductivity	Promoted Schwann cell proliferation and migration, proving to be a promising material for peripheral nerve regeneration.	2020 [163]
	Au-PCL-based nanolayer	In vitro/PC12, S42	Axonal growth Modulation of signaling pathway Electrical conductivity	It exhibited high electrical conductivity, promoting nerve cell growth and neurite regeneration, particularly under electrical stimulation (ES), making it a promising material for nerve repair.	2020 [164]
	Nerve conduit containing Au	In vitro/PC12, S42, MCF-7	Axonal growth Modulation of signaling pathway Electrical conductivity	Promoted neural growth and differentiation of PC-12 and S-42 cells, showing potential as a material for peripheral nerve regeneration while also applicable for cancer cell removal via photothermal effects.	2024 [110]
	Self-curling silk NGC incorporating a wireless electrical stimulator (SNGC-WES)	In vitro/PC12, primary dorsal root ganglion (DRG) neuron In vivo/Sprague–Dawley (SD) rats	Axonal growth Modulation of signaling pathway Electrical conductivity	Exhibited a notable improvement in axonal regeneration and functional recovery by enhancing mitochondrial anterograde transport.	2024 [76]
Ag	Nanosilver-collagen scaffold	In vivo/New Zealand rabbit	Neuroprotection Schwann cell myelination Axonal growth Controlled ion release	Enhanced laminin adsorption promotes nerve regeneration and improves axon growth and myelination, accelerating nerve function recovery.	2010 [113]
	PVA-based tubes mixed with MWCNTs or PPy	In vitro/primary hMSC	Electrical conductivity	The materials demonstrated electrical conductivity and were developed as promising biomaterials for nerve regeneration, and were evaluated for their potential in clinical applications	2016 [116]
Ag	nBG/Gel/nAg conduit	In vitro/*S. aureus*, *E. coli*, human airway fibroblast cell	Antibacterial activity Controlled ion release	Exhibited antimicrobial properties while maintaining low cytotoxicity, showing potential to reduce infection risks while supporting nerve regeneration	2019 [71]
	AgNWs/PDMS electrodes	In vitro/PC12	Axonal growth Modulation of signaling pathway Electrical conductivity	Electrical stimulation through these electrodes promoted nerve cell proliferation and axon growth, positioning this platform as a promising tool to study the effects of electro-mechanical stimulation on nerve regeneration	2021 [115]

#### Zinc ion

Zinc is an essential trace element crucial in peripheral nerve function and regeneration, particularly in neurogenesis [102,103]. It supports critical processes for nerve healing, such as cell division, survival, and proliferation. Zinc also supports axonal growth and myelination, both essential for effective nerve regeneration. Zinc-loaded biomaterials have been developed to exploit zinc’s peripheral nerve regenerative potential. These biomaterials release zinc ions to promote nerve repair and functional recovery or enhance peripheral nerve regeneration through electrical stimulation using zinc (Table 1).

Ekram et al. [65]​ incorporated zinc ions into electrospun PCL conduits for peripheral nerve regeneration (Fig. 3). These zinc-based biomaterials increase mechanical properties and stimulate mesenchymal stem cell proliferation. Zinc is bioactive by promoting cell attachment and axonal growth while offering antibacterial effects, particularly against *Staphylococcus aureus*. The controlled release of zinc through PCL conduits enhances the antibacterial and nerve regeneration. Yu et al.​ [11] developed a fully biodegradable, self-powered NGC based on dissolvable zinc-molybdenum batteries. These NGCs provide continuous electrical stimulation to support Schwann cell proliferation and ATP production, both vital for nerve repair. Zinc ions enhance axonal regrowth and remyelination, promoting functional recovery. This biodegradable biomaterial utilizes zinc batteries to provide sustained electric fields to improve therapeutic efficacy in rodents’ sciatic nerve defects. Zhang et al. [104] introduced an innovative self-powered microneedle-guided nerve conduit (MNGC) to enhance sciatic nerve repair. The MNGC consists of a polydopamine-modified polypyrrole/polyvinyl alcohol (DPP) hydrogel and enzyme-loaded nanoparticles [glucose oxidase (GOx) and horseradish peroxidase (HRP)] in a microneedle (MN) array. These components enable an enzymatic cascade reaction that generates microcurrents from blood glucose, promoting axonal outgrowth, myelination, and muscle regeneration. The MNGC demonstrated excellent biocompatibility and stability, sustaining its mechanical properties for 12 weeks in vivo. Qian et al. [66] used a piezoelectric scaffold composed of PCL integrated with ZnO nanogenerators for peripheral nerve repair. Fabricated using 3-dimensional (3D) multilayer fabrication, the scaffold leverages ZnO’s piezoelectric properties to generate electrical signals from mechanical deformation, creating a bioelectric microenvironment that enhances neural regeneration. The scaffold demonstrates favorable mechanical properties, such as high elasticity, aligned porosity, and improved wettability, facilitating Schwann cell adhesion and proliferation. In vitro studies reveal that Schwann cells on the ZnO/PCL scaffold exhibit enhanced viability, proliferation, and expression of neural (MBP, Tuj1) and vascular (VEGF) markers under piezoelectric stimulation. In vivo, the scaffold was tested in a rat sciatic nerve injury model with a 15-mm defect. Mechanical stimulation through treadmill exercise-induced bioelectric currents significantly improves axon remyelination, nerve conduction velocity, and motor function recovery. The ZnO/PCL scaffold outperformed a nonpiezoelectric PCL counterpart and approached the regenerative performance of autografts. However, relatively limited research has been conducted to develop a new approach for peripheral nerve regeneration using zinc-directed biomaterials. Furthermore, work is still needed to understand their therapeutic effects fully.

### Implantable metal

#### Iron ion

Iron is essential for peripheral nerve regeneration. It is critical in biological processes such as oxygen transport, DNA synthesis, and neurotransmitter production [105]. Iron transport proteins like DMT1 and transferrin receptor 1 are necessary for proper myelin production and maintenance [106]. Nerve injury increases iron uptake at the injured site, favoring Schwann cell differentiation and nerve repair. Iron-based biomaterials leverage the regenerative properties of iron by utilizing the controlled release of iron ions and magnetic fields to support regeneration in cases of PNI (Table 1). Azzouz et al. [107] reported that a solution of iron sulfate (FeSO_4_) has a protective effect against demyelination and promotes nerve repair. In injured frog sciatic nerves, these biomaterials have been shown to reduce myelin degradation and increase Schwann cell proliferation. Iron could prevent degeneration or promote regeneration induced by ligation in injured nerves. Biomaterials like magnetic fibrin nanofiber hydrogels loaded with iron oxide nanoparticles (IONPs) have been used to promote peripheral nerve regeneration (Table 1), incorporating iron ions (Fe^3+^), according to Hong et al.​ [108]. These hydrogels sustain Fe^3+^ and electrical stimulation, enhancing Schwann cell proliferation and supporting myelination. IONPs revealed a remarkable increase in NGF and VEGF expression in PC12 cells. Combining iron ions and fibrin hydrogels also accelerates axonal regeneration and functional recovery, showing great potential for peripheral nerve repair. According to Liu et al. [30], iron ions (Fe^3+^) are incorporated into SPIONs used in biomaterials for peripheral nerve regeneration. These SPIONs, when combined with magnetic actuation, stimulate Schwann cells to adopt repair-supportive phenotypes, promoting axonal regrowth and myelination. The magnetic forces generated by SPIONs enhance Schwann cell proliferation and nerve repair (Table 1). Liu et al. [81] developed a novel electroconductive hydrogel (ECH) dressing to treat diabetic PNIs, which often suffer from poor axon regeneration and severe demyelination. Comprising TA and polypyrrole (PPy), the ECH is thin, flexible, adhesive, and self-healing. It forms a tubular structure around injured nerves, ensuring close contact without invasive procedures. The hydrogel shows excellent mechanical and electrical properties and high biocompatibility. In vitro studies indicated enhanced Schwann cell adhesion, proliferation, and axonal outgrowth facilitated by the activation of the mitogen-activated protein kinase kinase (MEK)/extracellular signal-regulated kinase (ERK) signaling pathway. In diabetic rat models, the ECH significantly improved motor function recovery, nerve conduction velocity, and axonal remyelination over untreated controls. Histological analyses revealed well-organized nerve fibers and reduced muscle atrophy. The hydrogel exhibits minimal cytotoxicity and hemolysis, making it a safe clinical option. This innovative ECH dressing offers a noninvasive solution for diabetic PNIs, promoting nerve repair and functional recovery through bioelectrical stimulation and a biomimetic scaffold (Table 1). Xu et al. [29] developed injectable, electroconductive self-healing hydrogels and scaffolds that promote neural regeneration and motion sensing capabilities (Table 1). These hydrogels and scaffolds demonstrated excellent biocompatibility, biodegradability, and the ability to support the attachment, proliferation, and differentiation of neural stem cells (NSCs). In a zebrafish brain injury model, the NSC-laden conductive hydrogel significantly improved functional recovery compared to controls (Table 1). The genes involved in the process of neural regeneration include glial fibrillary acidic protein (GFAP) (glial marker), βIII-tubulin (early neuronal marker), and microtubule-associated protein 2 (MAP2, mature neuronal marker). These genes were significantly up-regulated in NSCs cultured in scaffolds composed of N-carboxyethyl chitosan (CEC), chitosan-modified polypyrrole (DCP), and difunctional polyurethane (DFPU), collectively referred to as CDD, after 14 d compared to the control group. Results indicated that CDD materials facilitate the survival, attachment, proliferation, and differentiation of NSCs. The presence of DCP nanoparticles in the CDD scaffolds may contribute to the proliferation of NSCs, promoting their differentiation into neurons. Iron-based biomaterials hold great potential for peripheral nerve regeneration by facilitating Schwann cell activity, enhancing myelination, and promoting axonal repair. Iron-containing biomaterials, including iron sulfate, IONPs, and magnetic hydrogels, have positively affected nerve regeneration and functional recovery. However, further research is needed to optimize iron ion delivery systems and fully explore their long-term therapeutic potential for nerve repair.

#### Gold ion

Gold is a promising candidate for peripheral nerve regeneration due to its high biocompatibility, electrical conductivity, and anti-inflammatory properties. It supports nerve repair by enhancing Schwann cell adhesion, proliferation, and migration, which are essential for nerve regeneration (Table 1). Additionally, gold’s electrical properties promote neurite growth and axonal extension. Gold nanoparticles (AuNPs) reduce oxidative stress and inflammation, creating a more favorable environment for nerve repair [109]. Gold-based biomaterials, such as AuNP-incorporated hydrogels and gold-coated scaffolds, are being explored to support nerve regeneration by biocompatibility and electrical support. Jaswal et al. [110] incorporated gold ions (Au^3+^) into polydopamine-coated gold nanoparticles (Au-PDA) embedded in PCL nanofibers for peripheral nerve regeneration. The AuNPs embedded in PCL scaffolds significantly enhance electrical conductivity, promoting peripheral nerve regeneration. Gold-incorporated nanofibrous scaffolds enhance Schwann cell adhesion, proliferation, and differentiation, which are key nerve repair factors [111]. Gold’s conductivity promotes neurite outgrowth and axonal regeneration, and the inclusion of AuNPs improves the scaffold’s mechanical properties, creating a favorable environment for nerve regrowth (Table 1). While gold-based materials show significant potential for peripheral nerve regeneration due to their biocompatibility and electrical conductivity, further research is required to explore their capabilities and fully address challenges in clinical application.

#### Silver ion

Silver-based biomaterials offer great potential for enhancing functional recovery in peripheral nerves. Silver-based biomaterials are an attractive option for peripheral nerve regeneration due to their nerve-regenerating properties through electrical conductivity (Table 1). Silver has strong antibacterial properties, which prevent infections during nerve regeneration [112]. This property is instrumental in maintaining a sterile environment around the regenerating nerve, reducing the risk of scaffold-associated bacterial infections [71,113]. This balance is critical for ensuring the safety and efficacy of the materials used in nerve regeneration. It reduces inflammation, creates a favorable environment for peripheral nerve regeneration, and promotes Schwann cell proliferation, migration, and axonal regrowth (Fig. 2G). Silver nanoparticles (AgNPs), incorporated into biomaterials like chitosan and PCL, guide nerve cell adherence while also providing an anti-infection effect [114]. These silver-based biomaterials also reduce inflammation, improving the conditions for nerve repair. However, controlling AgNP dosage and release is crucial to avoid cytotoxic effects during nerve regeneration. To solve the problem, silver ions (Ag^+^) have been incorporated into biomaterials, such as silver nanowires (AgNWs) embedded in polydimethylsiloxane (PDMS), and used as electrodes for peripheral nerve regeneration [115]. These AgNWs/PDMS scaffolds promote cell proliferation and axon outgrowth in a specific direction through electrical stimulation, which is essential for nerve repair. Scaffolds containing nanosilver have shown superior functionality in nerve regeneration. These scaffolds promote the adsorption of laminin, a protein essential for nerve growth, leading to improved nerve conduction velocity and thicker myelin sheaths in regenerated nerves [71]. Electrically conductive materials have been developed using a poly (vinyl alcohol) (PVA) matrix incorporated with various conductive agents, including nonfunctionalized multiwall carbon nanotubes (MWCNTs), PPy, silver nitrate (AgNO_3_), and magnesium chloride (MgCl_2_), to enhance nerve regeneration through conductive tube-guide applications [116]. The relationship between electrical conductivity and nerve regeneration could solve the limitations of existing nerve tube guides available. Moreover, the prospective results of silver-based electrodes for peripheral nerve regeneration demand further research on other conductive materials that can be combined with external stimulation for faster and better nerve regeneration.

### Other ions

Copper (Cu), silicon (Si), and cobalt (Co) ions are also used in biomaterials for peripheral nerve regeneration. Xu et al. [117] recently reported a copper ion-modified germanium-phosphorus (GeP) nanosheet integrated with a novel biohybrid biodegradable hydrogel (GelMA) for neuro-vascularized bone regeneration. This hydrogel exhibits effective antibacterial properties and promotes osteogenic differentiation, angiogenesis, and neural differentiation in vitro. In vivo studies demonstrated enhanced angiogenesis and neurogenesis in a rat calvarial bone defect model, contributing to improved bone regeneration. Neurogenesis markers, such as Tuj1 and MAP2, were significantly expressed in NSCs cultured on the GelMA/GeP@Cu hydrogel, indicating their involvement in neuronal differentiation. Nestin and GFAP were also examined, with lower expression levels observed in the presence of the GelMA/GeP@Cu hydrogel, suggesting a shift toward neuronal rather than glial differentiation. Sun et al. [118] prepared a fully bioresorbable and conductive nerve scaffold integrating N-type silicon (Si) membranes, which enhances nerve regeneration and functional recovery in rodent models with sciatic nerve injuries. The scaffold promotes the proliferation of Schwann cells and enhances calcium activity in dorsal root ganglion (DRG) neurons, leading to accelerated nerve regrowth. Compared to the control and autograft groups, the N-type group exhibited significantly less muscle atrophy and improved muscle fiber recovery. The scaffold’s slow degradation rate allows for sustained electrical cues, which are beneficial for nerve repair. Experimental results demonstrate accelerated nerve regeneration and improved motor function recovery in rodent models, highlighting the scaffold’s potential in regenerative medicine. Another study investigated the effects of cobalt chloride (CoCl_2_) on functional recovery following sciatic nerve transection injury in rats [119]. It highlights the activation of the hypoxia-inducible factor-1α (HIF-1α) pathway, which is crucial for nerve regeneration. Results showed that CoCl_2_ significantly improved functional recovery, increased expression of neurotrophic factors (GDNF, BDNF, NGF), and enhanced nerve conduction. The HIF-1 pathway is identified as a central pathway for sensing and responding to alterations in oxygen levels, playing a crucial role in peripheral nerve regeneration. The findings suggest that CoCl_2_ may be a potential therapeutic agent for promoting peripheral nerve regeneration.

## Challenges with Metal-Based Peripheral Nerve Regeneration

The use of metal-based biomaterials in peripheral nerve regeneration shows significant promise, but several key challenges must be addressed to unlock their complete regenerative potential [120]. These include biocompatibility, controlled ion release, mechanical properties, and material characteristic optimization for successful nerve regeneration. It is essential to develop these materials for biological compatibility and specific features, such as conductivity and long-term functionality.

### Long-term biocompatibility studies

It is known that a high concentration of metal nanoparticles in living organisms can cause cell oxidative stress and ROS production, leading to other severe cellular dysfunctions, such as inflammation, cell damage, DNA damage, cancer, or apoptosis [121]. While many metal ion-based scaffolds show promising biocompatibility, their long-term safety in physiological environments remains uncertain (Table 2). To address this, long-term in vivo studies must directly investigate scaffold integration with host tissues and monitor for any adverse reactions, such as inflammation, fibrosis, or tissue rejection [122]. Histological techniques are valuable for providing detailed insights into these tissue–scaffold interactions. Zhang et al. [10] demonstrated a concentration-dependent influence on the calcium titanate (CaTiO_3_) nanoparticle hybridized SF scaffolds. Optimal CaTiO_3_ nanoparticle concentration could stimulate the proliferation, attachment, and protection of Schwann cell biological functions. Nevertheless, the scarcity of in vivo investigations hampers the ability to evaluate possible immune responses and long-term degradation behaviors. Similarly, Yang et al. [96] express concerns about the long-term toxicity or accumulation of Li-CPDs in vital organs such as the kidneys and brain. The therapeutic window of existing lithium drugs is exceptionally narrow, and the adverse side effects severely limit their therapeutic value [123]. On a maximum of 21 d of experiments, in vivo, results confirmed that Li-CPDs promoted nerve regeneration by activating autophagy of Schwann cells and exhibited almost no renal toxicity. To assess biosafety, long-term studies are needed to verify the safety of Li-CPDs as they degrade and are cleared from the body. Finally, Hong et al. [108] emphasize that although magnetic fibrin nanofiber hydrogels demonstrate promising short-term biocompatibility, their rapid degradation could limit sustained support for nerve regeneration. Furthermore, the long-term presence of IONPs raises concerns about potential toxicity or chronic immune reactions. Extended in vivo testing is needed to ensure that the hydrogel and IONPs do not cause long-term inflammation or other toxic effects​.

**Table 2.** Advantages, considerations, and limitations of metal-based regenerative strategies for peripheral nerve injury

Type	Metal	Advantages	Consideration	Limitations
Biodegradable	Mg	Natural biodegradability Induction of bioactive signaling	Requirement for controlled degradation rate Potential for local pH shift	Rapid corrosion Hydrogen gas generation Cytotoxicity at high concentrations
	Li	Schwann cell activation Supports myelination Reduction of oxidative stress	Requirement for dose optimization Necessity of gradual and localized ion release	Inhibition of Schwann cell migration at high concentrations Risk of toxicity
	Ca	Well-established biocompatibility Dual function in signaling and structural support High compatibility with hydrogels and scaffolds	Necessity of controlled release systems Frequent requirement for combination with supportive materials (e.g., hydrogel, ceramics)	Potential cytotoxicity from excessive Ca^2+^ release Risk of inflammation without optimized formulation
	Li	Dual function as biochemical ion source and bioelectrical stimulator Support for mechanical strength and scaffold stability Antibacterial activity	Design complexity of piezoelectric or battery-based Zn systems Necessity for optimized degradation behavior	Lack of long-term in vivo evaluation Incomplete understanding of therapeutic mechanisms Risk of zinc toxicity at high concentrations
Implantable	Fe	Magnetic guidance capability for Schwann cells Possibility of electrical and mechanical stimulation	Requirement for external magnetic or electrical field control Dependency of efficacy on SPION design	Uncertainty in long-term biosafety of SPIONs Necessity for precise fabrication and field strength control
	Au	High biocompatibility Excellent electrical conductivity Anti-inflammatory properties	Requirement for optimized AuNP concentration Need for precise control of nanomaterial interactions	Uncertainty in long-term cytotoxicity High cost and complex fabrication protocols
	Ag	Strong antibacterial property Electrical stimulation-enhanced regeneration Anti-inflammatory effects	Requirement for optimized electrical and electromechanical stimulation Need for controlled ion release	Risk of cytotoxicity at high concentrations Uncertainty in long-term biosafety

### Controllable ion release

Optimum nerve regeneration necessitates controlled ion release from metal-based biomaterials. The uncontrolled release of excessive ions causes cytotoxicity and slows down nerve regeneration [124]. Establishing a controlled release condition is the most important target for researchers to achieve targeted healing. The fast degradation of metal ion-based materials might result in the loss of structural integrity and cause a sudden release of ions (Table 2). Furthermore, the nerve regeneration process may be hampered by gas pockets formed by the formation of hydrogen gas during the degradation of metal ions such as magnesium [125,126]. The release of magnesium ions must be carefully controlled to ensure sustained support throughout the nerve healing process and avoid additional tissue damage [127]. Applying protective coatings like poly (lactic-co-glycolic acid) (PLGA) or calcium phosphate, as well as developing magnesium alloys with elements like zinc and calcium, can slow degradation and allow for a more gradual ion release [128,129]. Gao et al.​ [16] discuss the difficulty in achieving controlled magnesium ion release from hydrogels, where an initial rapid release could lead to toxicity, while a slower release may fail to support nerve regeneration. Optimizing the release profile to match the body’s natural healing timeline is crucial. In another example, Yang et al. [130]​​ report that scaffolds incorporating calcium and magnesium show an initial burst of ion release followed by a slower, more stable phase. However, this early burst may not align with the slower pace of nerve regeneration, leading to potential imbalances in the bioactivity of the scaffold. Bu et al. [131] reported difficulty moderating lithium diffusion from their adhesive systems. While lithium release is sustained over time, ensuring the release rate aligns with the nerve regeneration process is crucial to avoid localized toxicity while maximizing therapeutic effects.

### Harmonious material integration

The integration process of metal-based transplantable materials is crucial for their success in nerve regeneration. The regenerative therapy must provide sufficient structural support while closely matching the niche of native nerve tissue to avoid complications like stress shielding [132]. A material, such as a nerve conduit, should have an elastic modulus similar to that of peripheral nerves, typically around 50 to 100 kPa, to ensure proper mechanical compatibility [133,134]. A rigid and stiff conduit causes substantial tissue damage and neuroinflammatory response. It impedes an animal’s natural behavior, whereas an excessively flexible conduit may not adequately support the regenerating nerves [135,136]. Magnesium-based biomaterials are widely used in biomedical applications because they are biodegradable and do not require secondary surgery to remove the implant [137,138]. Using histological and immunocytochemical analyses, magnesium metal filaments were observed to have good biocompatibility and only mild inflammation in tissues. However, pure magnesium has an average Young’s modulus of approximately 45 GPa [139], which can lead to inflammation and damage to surrounding tissues, potentially causing further nerve damage. To address this, alloys and composite materials have been developed to improve strength and flexibility. Composite scaffolds that combine metals with polymers or ceramics help balance mechanical resilience and biocompatibility. Hu et al. [140] reported that while adding reduced graphene oxide (rGO) to MN NGCs improved strength, the inclusion of ZnO nanoparticles introduced brittleness, highlighting the need for material optimization to maintain a balance between durability and flexibility. Similarly, Jaswal et al. [110] found that the Au-PDA integrated PCL scaffold demonstrated good initial mechanical properties, but its long-term resilience was a concern. Throughout the nerve regeneration process, reinforcement or material optimization may assist in guaranteeing that the material maintains its mechanical stability.

### Sustained electrical stimulation

Maintaining stable conductivity is essential for the success of electrically conductive materials in nerve regeneration [141]. Electrical stimulation is critical in promoting nerve cell growth and enhancing repair. However, ensuring consistent conductivity throughout the healing process remains a significant challenge for many metal-based biomaterials (Table 2) [142]. These conductive materials must provide electrical stimulation initially and maintain it over time as the material degrades or experiences mechanical stress. For instance, Lee et al. [143] explored magnesium combined with molybdenum to improve electrical conductivity for nerve stimulation. As promising as this combination is, the critical issue lies in durability over time and the loss of its conductive properties. Similarly, Yu et al. [11] noted that while demonstrating good initial electrical performance, the degradation of the biodegradable materials disrupted the electrical stimulation over time. This degradation can compromise the consistent delivery of electrical stimulation, which is crucial for sustained nerve regeneration. Xia et al. [144] also emphasized the difficulty of maintaining stable conductivity in superparamagnetic scaffolds, mainly due to the uneven distribution of magnetic nanoparticles. Inconsistencies in conductivity can create localized areas with reduced conductivity, which decreases the efficiency of nerve stimulation. Additionally, as the transplanted material degrades over time, its overall conductivity may diminish, negatively impacting nerve regeneration in the long term. Lastly, Xu et al. [115] highlighted that while AgNWs embedded in PDMS electrodes initially exhibit good conductivity, repeated mechanical stretching during use can lead to increased resistance, reducing the effectiveness of electrical stimulation. To ensure consistent conductivity throughout the stretching process, future research should focus on minimizing resistance fluctuations by optimizing electrode design and properties of different materials​.

## Comparison of Biodegradable and Implantable Metal Strategies

Biodegradable and implantable metal-based strategies are capable of effective peripheral nerve regeneration. A comparative assessment of these strategies is essential to guide rational material selection for multifaceted regenerative therapies. Biodegradable metals, such as magnesium (Mg)-, zinc (Zn)-, and iron (Fe)-based alloys, are designed to undergo progressive degradation in physiological environments, releasing bioactive ions that can influence cellular processes, including proliferation, differentiation, angiogenesis, and axonal regeneration [14]. These ions not only serve as degradation products but also actively participate in orchestrating regenerative cascades. Conversely, while largely stable, implantable metals such as gold (Au) and silver (Ag) have demonstrated electrical and electromagnetic properties that can modulate gene expression, synaptic activity, and neurogenesis when integrated into conductive scaffolds [111,112].

The trade-offs between these strategies are evident. Biodegradable metals offer temporal bioactivity and absorbability, making them ideal for transient applications such as nerve conduits, bioresorbable stents, or scaffolds for soft tissue regeneration. In contrast, nondegradable implants are preferred in contexts demanding permanent structural support, such as spinal fixation or load-bearing joint replacements. Importantly, emerging hybrid systems, such as core–shell designs that combine a bioresorbable metal core with a stable metal or polymeric shell, aim to synergize the advantages of both strategies.

While metallic ions offer therapeutic benefits in nerve regeneration, several limitations constrain their clinical application. Magnesium degrades rapidly, releasing hydrogen gas and causing cytotoxicity at high concentrations. Lithium has a narrow therapeutic window, with risks of renal toxicity and inhibition of Schwann cell migration. Calcium may induce cytotoxicity and inflammation if not precisely controlled, often requiring supportive biomaterials. Zinc systems are complex in design, with mechanisms still under investigation. Iron (Fe^2+^) can generate harmful ROS, while Fe^3+^-based systems depend on external field control and raise biosafety concerns [145]. Gold and silver nanoparticles face issues of cytotoxicity, high cost, and uncertain long-term safety, necessitating tight control over dosage and material interactions (Table 2).

**Fig. 4.**
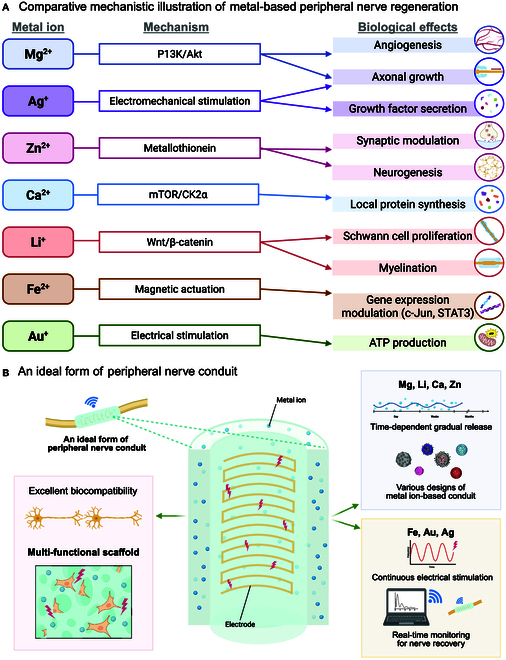
(A) Comparative mechanistic illustration of metal-based peripheral nerve regeneration and an ideal form of peripheral nerve conduit. The relationship between representative metal ions, their associated molecular mechanisms, and the resulting biological effects in the context of peripheral nerve regeneration. The visual layout enables cross-comparison of how different ions contribute to key processes such as axonal growth, Schwann cell proliferation, and myelination through distinct mechanistic pathways. (B) Illustration of an ideal metal-based nerve conduit. An ideal peripheral nerve conduit should be excellent biocompatible, capable of releasing metal ions gradually, and long-term conductive to stimulate nerve cells when necessary.

In summary, the comparative application of biodegradable versus nondegradable metal biomaterials should be driven by a nuanced understanding of mechanical requirements, desired degradation kinetics, and targeted cellular responses. Furthermore, integrating metal-based systems with electrical stimulation platforms or innovative bioelectronic interfaces opens new avenues for controlled cell modulation. As illustrated in the mapping, each metal ion derived from degradation or surface coating can interact with specific intracellular signaling pathways, providing a modular approach to engineering bioactivity (Fig. 4A).

## Future Directions and Perspectives

A detailed understanding of metal-based biomaterials for peripheral nerve regeneration must prioritize long-term biocompatibility to ensure their successful application in nerve regeneration (Fig. 4B). Long-term in vivo studies should evaluate critical factors, such as the material’s degradation rates (time kinetic metal ion levels in plasma and urine), immune responses due to prolonged metal ion exposure (both pro-inflammatory and auto-inflammatory), and time-dependent tissue integration of metal ions (long-term histopathological analysis). These studies will provide essential data for optimizing materials for translating into clinical applications with a broad and safe window. Next, optimizing ion release profiles is crucial for the effectiveness of metal ion-based scaffolds. Research should focus on developing materials with different combinations of one or more metal ions to investigate the tissue healing timeline that allows sustained, controlled ion release. The investigation should ensure that the bioactive benefits of metal ions are utilized without compromising biocompatibility. Improvement of the mechanical properties of metal ion-based materials is needed to promote nerve regeneration effectively. Comprehensive studies should be conducted to achieve an optimal elastic modulus, enhance durability, and maintain flexibility through various material combinations. This will ensure that the material provides consistent support without causing a mismatch with surrounding tissues. Furthermore, the resulting material should also exhibit stable metal ion-dependent conductivity for long-term nerve regeneration. Advanced peripheral nerve regenerative materials should enhance the uniformity of resistance and durability of conductive properties to enable effective electrical stimulation over extended periods, for example, incorporating more stable conductive materials, such as molybdenum, or designing hybrid systems that maintain electrical performance throughout the therapeutic period. Ensuring extensive research to address all the problems mentioned earlier could lead to the development of optimal nerve-regenerating materials that replace autograft transplantation and produce marketable, universal products.

## Conclusion

Metal-based biomaterials are crucial in regulating peripheral nerve regeneration, influencing axonal growth, Schwann cell activity, and modulation of inflammation. Despite their potential, challenges such as optimizing long-term biocompatibility, controllable ion release, and balancing mechanical properties and electrical conductivity remain to be addressed. Addressing these issues will be critical to realizing the full therapeutic potential of metal-based tissue regeneration for PNIs. However, utilizing biofunctional ions with biomaterials decreases costs and stem cell-related complications, and increases control over the treatment scheme. Biofunctional metal ions offer a promising strategy to enhance peripheral nerve regeneration, addressing the limitations of current nerve repair techniques and paving the way for advanced therapeutic interventions.

## Review Methodology

This study highlights the crucial role of biofunctional metal-based tissue engineering and regenerative medicine in peripheral nerve regeneration. To conduct this review, we searched PUBMED with keywords like “Peripheral nerve injury”, “Metal ion (e.g., Magnesium, Lithium, Calcium, Zinc, Gold, Iron, Silver, Cobalt, Copper, Silicon)”, “Regenerative medicine”, or “Biomaterials”. We extensively researched relevant scholarly articles published between January 2010 and December 2024. We only considered articles written in English and published in peer-reviewed journals. Research articles aimed to treat PNI with tissue engineering and regenerative medicines were considered.
